# The pathways to teacher growth: mediating roles of motivation and self-efficacy within the community of inquiry framework

**DOI:** 10.3389/fpsyg.2026.1755265

**Published:** 2026-03-24

**Authors:** Mingzhang Zuo, Dengfeng Yang, Lei Xiang, Zhimiao Yang

**Affiliations:** 1 Faculty of Artificial Intelligence in Education, Central China Normal University, Wuhan, China; 2 School of Education, China West Normal University, Nanchong, China

**Keywords:** community of inquiry, learning motivation, rural teacher, self-efficacy, sense of immediacy, teacher professional development

## Abstract

**Introduction:**

Teacher professional development is a key factor in enhancing the quality of education. Within technology-mediated or structured training environments, the concept of “presence”—particularly as defined by the Community of Inquiry (CoI) framework—offers a valuable lens for understanding how different types of interactions shape learning experiences. However, the specific ways in which these dimensions of presence can be translated into tangible professional development outcomes for teachers remain unclear.

**Methods:**

This study employed structural equation modeling to examine how different dimensions of presence shape teachers’ professional development outcomes, focusing on the mediating roles of learning motivation and self-efficacy. Data were collected from 1,298 teachers who participated in the training from rural schools in the Chengdu-Chongqing region and from a few universities in Sichuan Province.

**Results:**

The results indicate that teaching presence positively predicts learning motivation and cognitive presence but not self-efficacy or professional development outcomes. Social presence significantly predicts self-efficacy and cognitive presence yet shows no significant effects on learning motivation or professional development outcomes. In contrast, cognitive presence exerts significant positive effects on learning motivation, self-efficacy, and professional development outcomes. Moreover, learning motivation and self-efficacy do not mediate the effects of teaching presence or social presence on professional development, but they do significantly mediate the relationship between cognitive presence and professional development outcomes.

**Discussion:**

These findings suggest that the pathway from cognitive presence to professional development outcomes, mediated by motivation and self-efficacy, constitutes the central mechanism of teacher growth. Teaching presence primarily facilitates cognitive construction and motivation, whereas social presence functions as a supportive safeguard through community building and efficacy enhancement. Overall, the study underscores that teachers, as adult learners, are more strongly driven by cognitive fulfillment and competence development than by social interaction or emotional support.

## 1 Introduction

Teachers’ professional development (TPD) is widely recognized as a cornerstone of educational improvement and innovation. With the rapid expansion of blended and online learning environments, it has become increasingly important to clarify the mechanisms that shape the effectiveness of professional learning. Compared with traditional face-to-face programs, blended contexts require teachers to engage in more complex interactions across pedagogical design, social engagement, and cognitive processes, all of which jointly influence the quality of their professional development experiences. It is essential to note that teachers involved in professional development are typical adult learners, and their learning process is different from that of students. They construct knowledge and skills based on rich experience, have a strong desire for autonomous learning, and always pursue the ability to solve practical problems (Knowles et al., 2014; Laurian-Fitzgerald et al., 2018; Zhang et al., 2025). This perspective on adult learning requires special consideration when applied to professional development training.

The Community of Inquiry (CoI) framework (Garrison et al., 1999) provides a widely validated lens for analyzing these interactions. Within this framework, teaching presence, social presence, and cognitive presence constitute essential conditions for meaningful engagement: teaching presence ensures structure and facilitation, social presence fosters collaboration and emotional connectedness, and cognitive presence drives inquiry and knowledge construction. While prior studies have demonstrated the value of the CoI framework in explaining teacher learning in blended environments (Vaughan et al., 2013), the specific pathways through which different forms of presence influence TPD outcomes remain insufficiently specified.

At the same time, individual psychological mechanisms are central to sustaining teacher learning. Learning motivation, emphasized in self-determination theory (Deci and Ryan, 2000), functions as a key driver of persistence and achievement, whereas self-efficacy, grounded in social cognitive theory (Bandura, 1997), predicts effort, resilience, and performance. Both constructs are recognized as critical determinants of teachers’ professional growth. Yet, the extent to which motivation and self-efficacy mediate the relationships between presence and TPD outcomes has not been systematically examined.

Although presence, motivation, and self-efficacy have each been acknowledged as influential, few studies have investigated their interplay within a unified model (Akcaoglu and Akcaoglu, 2022). Empirical evidence on how teaching, social, and cognitive presence affect TPD outcomes through the mediating roles of learning motivation and self-efficacy remains limited (Bardach et al., 2022). Addressing this gap is essential not only for enriching theoretical models of teacher learning but also for informing the design, implementation, and evaluation of professional development initiatives that leverage presence to enhance teachers’ engagement and growth.

Based on these considerations, this study employed the structural equation model (SEM) and combined three dimensions of presence, motivation and self-efficacy as mediating variables to test the comprehensive model. The main objective is to investigate the specific impact paths of teaching, social and cognitive factors on the outcomes of teachers’ professional development, and to determine the mediating roles of learning motivation and self-efficacy in these relationships. Theoretically speaking, this work provides a mechanism-based understanding for teacher learning by integrating the community inquiry framework with key psychological concepts. From a practical perspective, the research results aim to provide evidence-based guidance for designing effective professional development plans in a blended learning environment.

## 2 Literature review

### 2.1 Community of inquiry (CoI)

The Community of Inquiry (CoI) framework provides a robust theoretical foundation for understanding how teaching, social, and cognitive presences collectively shape meaningful learning in online and blended professional development (Garrison et al., 1999; Kozan and Caskurlu, 2018). However, significant gaps remain when applying this framework specifically to teacher professional development outcomes (PDO). Firstly, most CoI studies are based on students’ learning contexts; the specific paths and effectiveness of these studies in the context of teacher professional development have not been fully explored yet (Jia and Li, 2020; Guo et al., 2024). Secondly, Teacher motivation and self-efficacy are also often discussed as important outcomes for professional development, there is still a lack of research that integrates these two factors as mediators and places them within the CoI framework for teacher blended professional development. Current research often either focuses on the direct impact of CoI (Li and Jia, 2025), or examines motivation or self-efficacy as an independent mediating variable in traditional contexts (Xie and Wang, 2024; Xie and Shi, 2024), but fails to integrate it into the specific influence path mediated by CoI for teachers. Thirdly, Previous studies on the direct impact of teaching and social presence on learning outcomes have yielded inconsistent and sometimes insignificant results. This inconsistency suggests that the influence might be more indirect and mediated by other factors. To rigorously test this possibility, we will conduct mediating analysis using the structural equation model (Martin et al., 2022; Sun and Yang, 2023). From a theoretical perspective, setting motivation and self-efficacy as mediating variables stems from the integration and application of social cognition theory and self-determination theory. The three aspects of CoI’s existence do not directly translate into improvements in teaching practice; instead, they first have an impact on teachers’ inner psychological states and motivational systems (Germani, 2024). It is precisely this activated internal driving force and belief that ultimately drives the transformation of the acquired knowledge into a continuous teaching revolution. Therefore, this study aims to fill these gaps by specifically examining the mediating role of learning motivation and self-efficacy in the connection between CoI and the PDO pathway, thereby revealing the internal psychological mechanism through which CoI promotes teachers’ professional growth.

Based on this, this research raises the following three research questions:

Q1. Do the aspects of teaching presence, social presence and cognitive presence affect the outcome of teachers’ professional development?

Q2. What is the role of teachers’ learning motivation and self-efficacy in mediating the process?

Q3. When predicting the teachers’ professional development outcomes, what are the relative predictive powers of teaching presence, social presence, and cognitive presence?

Teaching presence refers to the design, facilitation, and direction of cognitive and social processes to achieve learning outcomes that are personally meaningful and educationally worthwhile. It encompasses three main functions: instructional design and organization, facilitating discourse, and direct instruction (Anderson et al., 2001). A well-designed and effectively guided instructional process can significantly foster the development of social presence and deepen cognitive presence (Garrison and Cleveland-Innes, 2005). The three dimensions of presence within the Community of Inquiry framework are significantly and positively correlated with learners’ actual academic achievement and soft skills (Tusyanah et al., 2023). Many studies highlighted the use of COI to assist learners in achieving learning VALUEs, such as improving students’ learning skills, promoting critical thinking and improving students’ academic performance (Gao et al., 2023). Teaching presence can facilitate cognitive presence. Especially for progressing to the higher-order resolution stage requires more deliberate instructional design and guidance in terms of teaching presence (Shea et al., 2010). However, some scholars have raised doubts. Research has indicated that there is no conclusive evidence that a Community of Inquiry can provide learners with deep and meaningful learning outcomes (Rourke and Kanuka, 2009). Literature reviews indicate that the critical link between presence and actual learning outcomes still lacks solid evidence and requires further validation (Martin et al., 2022). Therefore, the following hypotheses are proposed:

*H1*: Teaching presence positively predicts teacher professional development outcomes.

*H2*: Teaching presence positively predicts cognitive presence.

*H3*: Teaching presence positively predicts teachers’ learning motivation.

*H4*: Teaching presence positively predicts teachers’ self-efficacy.

Social presence refers to teachers’ ability to present themselves as “real people” in a collaborative community, fostering trust, rapport, and supportive interpersonal relationships (Garrison, 2009). Social presence is considered an important mediator for the development of cognitive presence. A community atmosphere characterized by mutual trust and open communication provides the climate for participants to engage in critical reflection and discourse (Akyol and Garrison, 2008). A high-level Community of Inquiry fosters a more engaging and supportive learning environment, as demonstrated by the correlation between cognitive presence and improved outcomes (Akyol and Garrison, 2011) and the consistent link between stronger overall presence perceptions and higher performance (Rockinson-Szapkiw et al., 2016). High social presence encourages collegial interaction, exchange of pedagogical experiences, and mutual support, creating an environment conducive to engagement. Consequently, social presence is expected to directly influence professional development outcomes, cognitive presence, learning motivation, and self-efficacy. Social presence functions as an emotional and relational foundation that indirectly facilitates teachers’ confidence and sustained participation. Accordingly, the following hypotheses are proposed:

*H5*: Social presence positively predicts teacher professional development outcomes.

*H6*: Social presence positively predicts cognitive presence.

*H7*: Social presence positively predicts teachers’ learning motivation.

*H8*: Social presence positively predicts teachers’ self-efficacy.

Cognitive presence reflects the extent to which teachers construct and confirm meaning through reflective inquiry and dialog (Garrison et al., 2001). Operationalized through the Practical Inquiry Model—triggering events, exploration, integration, and resolution—cognitive presence enables critical examination of teaching practices and application of innovative strategies. Higher cognitive presence is associated with enhanced professional development outcomes, learning motivation, and self-efficacy, forming the intellectual core of the CoI framework. Thus, the following hypotheses are proposed:

*H9*: Cognitive presence positively predicts teacher professional development outcomes.

*H10*: Cognitive presence positively predicts teachers’ learning motivation.

*H11*: Cognitive presence positively predicts teachers’ self-efficacy.

### 2.2 Learning motivation

Learning motivation refers to the internal psychological processes or drives that directly initiate, guide, and sustain individual learning activities (Pintrich and Schunk, 2002). As an internal force driving engagement in learning activities and sustaining learning behaviors, learning motivation profoundly influences the breadth, depth, and persistence of teachers’ participation in professional development (Richter et al., 2011). Teachers’ professional learning motivation is rooted in their complex professional roles and workplace contexts. As mature learners and professionals, their motivation exhibits the following distinct characteristics: strong career orientation (Guskey, 2002), inherent social altruism (Butler, 2007), deep connection with professional identity, and high sensitivity to organizational factors (Fullan, 2007). The satisfaction of autonomy, competence, and relatedness needs all contribute to learning motivation, teachers who can choose learning topics, experience success in applying new teaching strategies, and engage in supportive professional communities exhibit higher motivation (Deci and Ryan, 2000; Van den Berghe et al., 2014; Vangrieken et al., 2015; Tschannen-Moran and Woolfolk Hoy, 2001; Vescio et al., 2008; Kwakman, 2003; Guskey, 2002; Little, 1982).

Intrinsic motivation has been identified as a key predictor of teachers’ participation in online professional development and their active learning engagement (Gorozidis and Papaioannou, 2014). Learning motivation directly impacts professional development outcomes by increasing active participation, reflection, and adoption of innovative instructional practices. Moreover, it serves as a mediator between CoI presences and outcomes: teaching presence provides structure and guidance that enhance motivation; social presence offers relational support and recognition that reinforce engagement; cognitive presence provides intellectual challenge and opportunities for meaningful problem-solving that stimulate motivation. Therefore, the following hypotheses are proposed:

*H12*: Learning motivation positively predicts teacher professional development outcomes.

*H13*: Learning motivation mediates the relationship between teaching presence and professional development outcomes.

*H14:* Learning motivation mediates the relationship between social presence and professional development outcomes.

*H15*: Learning motivation mediates the relationship between cognitive presence and professional development outcomes.

### 2.3 Self-efficacy

Self-efficacy refers to an individual’s beliefs and judgments regarding their capability to organize and execute specific actions and successfully achieve intended goals (Bandura, 1997). It is influenced by mastery experiences, vicarious experiences, social persuasion, and emotional states. Teachers with high self-efficacy are more likely to adopt innovative teaching methods, persist through challenges, and create supportive learning environments, which in turn enhance professional development outcomes (Tschannen-Moran et al., 1998; Ashton and Webb, 1986; Guskey, 1988; Woolfolk Hoy and Burke-Spero, 2005; Klassen and Tze, 2014; Zee and Koomen, 2016; Skaalvik and Skaalvik, 2010; Brouwers and Tomic, 2000; Tschannen-Moran and Woolfolk Hoy, 2007; Thoonen et al., 2011).

The CoI presences support self-efficacy in multiple ways. Teaching presence provides mastery and guided experiences that strengthen confidence in instructional abilities. Social presence offers encouragement and recognition from peers, fostering self-belief. Cognitive presence engages teachers in reflective problem-solving, enhancing their perceived competence. Self-efficacy also positively reinforces learning motivation, forming a dynamic feedback loop that amplifies engagement and professional growth. Therefore, the following hypotheses are proposed:

*H16*: Self-efficacy positively predicts teacher professional development outcomes.

*H17*: Self-efficacy positively predicts learning motivation.

*H18*: Self-efficacy mediates the relationship between teaching presence and professional development outcomes.

*H19*: Self-efficacy mediates the relationship between social presence and professional development outcomes.

*H20*: Self-efficacy mediates the relationship between cognitive presence and professional development outcomes.

### 2.4 Conceptual model

**Figure 1:**
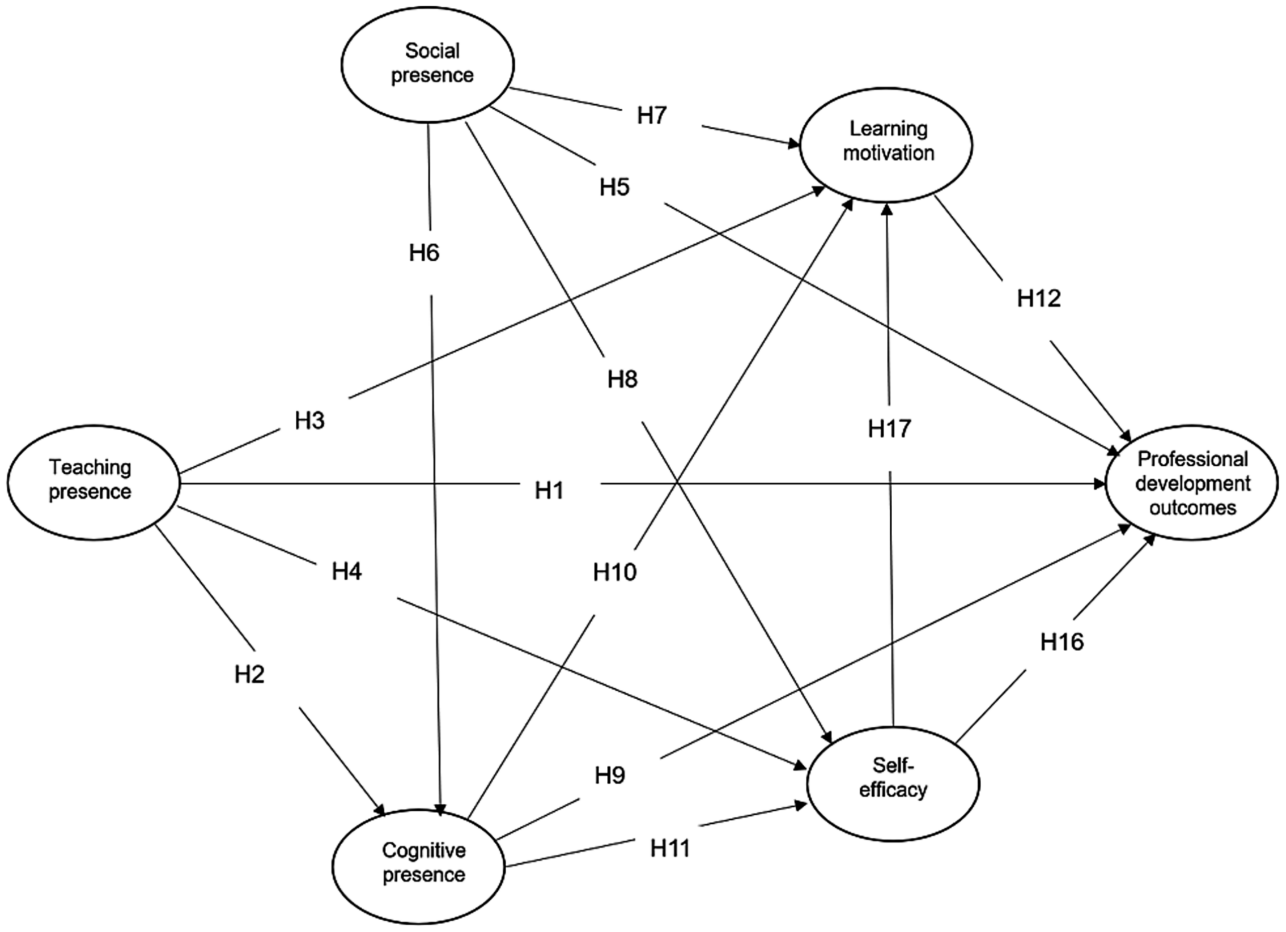
Research model (CP = cognitive presence, SP = social presence, TP = teaching presence, SE = self-efficacy, LM = learning motivation, PDO = professional development outcomes).

Based on the above review, teaching, social, and cognitive presences are hypothesized to influence teacher professional development outcomes both directly and indirectly through learning motivation and self-efficacy. Figure 1 illustrates the structural model corresponding to hypotheses H1–H20.

## 3 Methods

### 3.1 Participants and procedure

A quantitative research design was employed using a cross-sectional survey to collect data. The participants were teachers from rural schools in the Chengdu-Chongqing region and a small number of local universities in Sichuan Province, all of whom had engaged in professional development (PD) programs, including ongoing or recently completed workshops, training sessions, or online learning communities. The sample primarily consisted of K–12 teachers, with a smaller proportion of higher education instructors who had participated in teacher training programs. Efforts were made to ensure diversity in teacher characteristics and school contexts to enhance the precision of statistical analyses and the external validity of the findings.

Data were collected through an online questionnaire distributed via the Questionnaire Star platform using a snowball sampling method. A total of 1,920 responses were received. To ensure data quality, questionnaires with insufficient completion time or duplicate responses, which indicated potential careless answering, were excluded from analysis. After data cleaning, 1,298 valid responses remained, yielding an effective response rate of 67.6%. The lower response rate may reflect participants’ perception of the survey as an additional task, potentially leading to reduced engagement. Rigorous data cleaning procedures were applied to ensure the reliability of the results. Demographic characteristics of the sample are presented in Table 1.

**Table 1:** Detailed classification of demographic information.

Variable	Category	%of respondents (N = 1,298)
Gender	Male	31.5
	Female	68.5
Teaching experience	≤1 year	7.8
	2–5 years	12.5
	6–10 years	16.8
	11–20 years	31.3
	≥21 years	31.7
School type	Primary	78.3
	Secondary	16.6
	Higher Education	5.1

### 3.2 Research instruments

Data were collected via a structured online questionnaire administered through the Wenjuanxing platform, with distribution facilitated via teacher professional development project–specific social platforms and support from educational administrative bodies and participating schools. The study protocol and questionnaire received ethical approval from China West Normal University and relevant local education authorities.

The questionnaire comprised two sections. The first section collected demographic information. The second section, based on a 5-point Likert scale (1 = strongly disagree, 5 = strongly agree), measured four key constructs: CoI presence, learning motivation, self-efficacy, and teacher professional development outcomes.

The CoI presence was measured across three dimensions: teaching presence, social presence, and cognitive presence. Teaching presence included eight subdimensions: instructional goal setting, guidance of learning activities, development pathway planning, exploration of new concepts, focus on key issues, awareness of peer interaction, highlighting critical content, and instructional feedback and evaluation (8 items). Social presence covered seven subdimensions: community interaction, peer trust, peer respect, sense of belonging, community mediation, peer collaboration, and community awareness (7 items). Cognitive presence included seven subdimensions: problem awareness, knowledge transfer, problem solving, knowledge mastery, critical thinking, knowledge assessment, and meaning construction (7 items). Items were adapted from the CoI survey (Garrison et al., 2010; Arbaugh et al., 2008) to align with the teacher professional development context.

Learning motivation was assessed using a teacher-specific motivation scale targeting intrinsic and extrinsic motivation (8 items; Lin et al., 2020). Self-efficacy was measured using an established scale covering confidence, engagement, affect, problem solving, metacognition, and self-awareness (6 items; Schwarzer, 1993). Teacher professional development outcomes were measured across four subdimensions: instructional efficacy, professional reflection, professional autonomy, and innovative teaching behaviors, using 15 items adapted from Korthagen’s reflection scale and Berliner’s skill acquisition model (Korthagen, 2001; Hatton and Smith, 1995; Berliner, 2001).

### 3.3 Data analysis

To assess the reliability and validity of the questionnaire, confirmatory factor analysis (CFA) was conducted, with the results presented in Table 2. The Cronbach’s alpha coefficients for all constructs exceeded 0.9, indicating excellent reliability. The item loadings for each construct ranged from 0.732 to 0.938, meeting the standards for content validity (Doo and Bonk, 2020). The minimum average variance extracted (AVE) across all items was 0.65, exceeding the recommended threshold of 0.50 (Fornell and Larcker, 1981), indicating that the measurement model exhibits good convergent validity (Table 3), the correlation coefficients among the variables were all in the expected directions and statistically significant. Discriminant validity requires that the square root of the AVE for each construct exceeds the correlations between that construct and all other constructs (Gefen et al., 2000). In this study, except for a few correlation coefficients slightly below the square root of the AVE, the average variance extracted (AVE) values for all variables exceeded the absolute values of their correlations with other variables, indicating good discriminant validity of the measurement model. The confirmatory factor analysis and the interrelationships among variables statistically support the testing of the study’s hypotheses and the examination of mediating effects.

**Table 2:** Reliability and validity of the core constructs of the questionnaire.

Constructs	Number of items	Mean	Cronbach’s alpha	Factor loading	CR	AVE
TP	8	3.898	0.982	0.912–0.950	0.982	0.873
SP	7	3.963	0.971	0.850–0.938	0.971	0.829
CP	7	3.962	0.970	0.876–0.921	0.970	0.824
SE	6	3.916	0.967	0.832–0.915	0.957	0.790
LM	8	3.897	0.962	0.732–0.859	0.941	0.667
PG	15	3.947	0.974	0.734–0.857	0.965	0.648

**Table 3:** Two-tailed correlations among all constructs.

Constructs	Mean	SD	TP	SP	CP	SE	LM	PG
TP	3.8976	0.75271	0.934					
SP	3.9635	0.69991	0.877	0.910				
CP	3.9624	0.66596	0.843	0.876	0.908			
SE	3.9162	0.68299	0.712	0.761	0.802	0.889		
LM	3.8966	0.67227	0.771	0.791	0.826	0.857	0.817	
PG	3.9471	0.61718	0.725	0.752	0.803	0.877	0.835	0.805

To control for common method bias, this study employed procedural remedies such as anonymous responses and psychological separation during data collection. Statistically, Harman’s single-factor test was conducted, revealing multiple factors, with the first factor accounting for 34.9% of the total variance, below the critical threshold of 40%. For further examination, the unmeasured latent method factor (ULMC) approach was employed. Structural equation modeling analysis indicated that, after including the common method factor, the model fit indices did not change significantly and the coefficients and significance levels of all hypothesized paths remained substantively unchanged (Δdf = 51; ΔIFI = 0.018; ΔRFI = 0.024; ΔNFI = 0.018; ΔTLI = 0.025; ΔCFI = 0.018; ΔRMSEA = 0.009). Therefore, common method bias did not pose a serious threat to the results of this study.

The assessment indicated that the hypothesized model demonstrated an acceptable fit with the data (*x*^2^ = 10210.291, df = 1,210, IFI = 0.907, RFI = 0.890, NFI = 0.896, TLI = 0.902, CFI = 0.907, RMSEA = 0.076) (Table 4).

**Table 4:** Structural model fit indices.

NFI	RFI	IFI	TLI	CFI	RMSEA	SRMR	DF	PCMIN/DF
0.896	0.890	0.907	0.902	0.907	0.076	0.05	1,210	8.438

## 4 Results

### 4.1 Structural model assessment and hypotheses testing

The analysis involved the use of stricter statistical thresholds (*p* < 0.01) along with consideration of effect sizes. The analysis revealed that teaching presence (*β* = 0.030, *p* = 0.049) and social presence (*β* = −0.032, *p* = 0.137) did not have significant direct effects on teachers’ professional development outcomes, indicating that Hypotheses H1 and H5 were not supported. These results suggest that the direct influence of teaching and social presences on professional development is limited. In contrast, cognitive presence demonstrated a significant positive effect on teachers’ professional development outcomes (*β* = 0.176, *p* < 0.001), providing empirical support for Hypothesis H9 and highlighting the critical role of cognitive engagement in fostering professional growth (Table 5).

**Table 5:** Statistical significance test results.

Hypotheses	Path	Estimate	S. E.	C. R.	*P*	Results
H1	PDO ← TP	0.030	0.015	1.966	0.049	N
H5	PDO ← SP	−0.032	0.021	−1.486	0.137	N
H9	PDO ← CP	0.176	0.037	4.759	***	Y
H3	LM ← TP	0.147	0.016	9.141	***	Y
H7	LM ← SP	0.051	0.023	2.200	0.028	N
H10	LM ← CP	0.230	0.040	5.772	***	Y
H4	SE ← TP	−0.018	0.020	−0.879	0.379	N
H8	SE ← SP	0.198	0.030	6.614	***	Y
H11	SE ← CP	0.682	0.047	14.438	***	Y
H2	CP ← TP	0.302	0.013	23.153	***	Y
H6	CP ← SP	0.547	0.016	33.175	***	Y
H12	PDO ← LM	0.175	0.033	5.374	***	Y
H16	PDO ← SE	0.548	0.031	17.422	***	Y
H17	LM ← SE	0.541	0.027	20.359	***	Y

***indicates a highly significant result.

**Figure 2:**
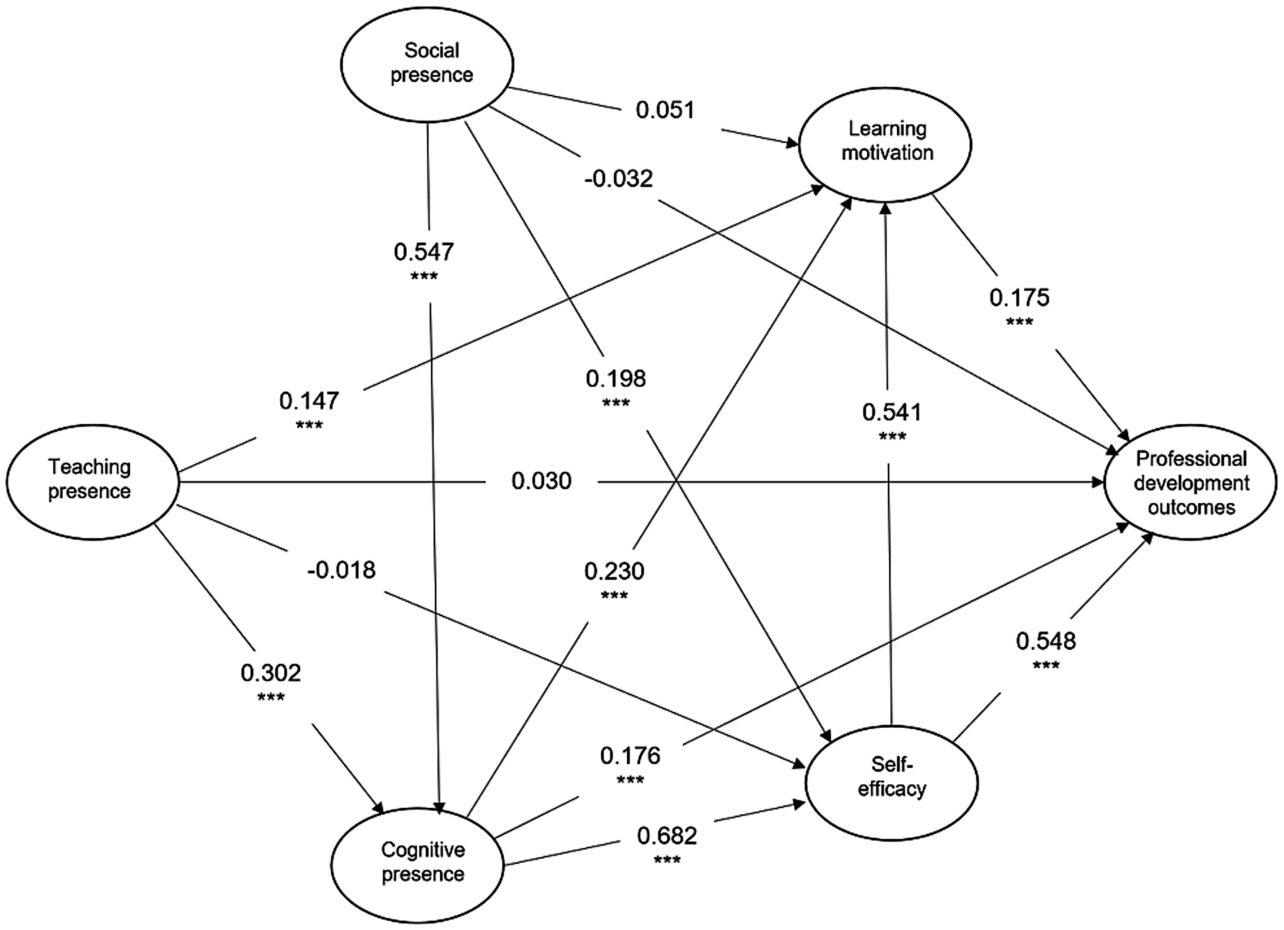
Structural equation model test results. ***indicates a highly significant result.

Regarding self-efficacy, the results indicated a strong and significant direct effect on professional development outcomes (*β* = 0.548, *p* < 0.001), confirming that teachers’ belief in their instructional capabilities directly enhances their professional growth. Similarly, learning motivation exhibited a significant positive effect on professional development outcomes (*β* = 0.175, *p* < 0.001), although its direct influence was smaller than that of self-efficacy (Figure 2).

When examining the predictors of psychological mechanisms, teaching presence significantly predicted learning motivation (*β* = 0.147, *p* < 0.001) but did not significantly predict self-efficacy (*β* = −0.018, *p* = 0.379), suggesting that while instructional design and facilitation enhance teachers’ motivation to engage, they do not directly strengthen teachers’ confidence in their professional abilities. Social presence had a non-significant positive effect on learning motivation (*β* = 0.051, *p* = 0.028) but significantly predicted self-efficacy (*β* = 0.198, *p* < 0.001), indicating that supportive social interactions primarily reinforce teachers’ self-beliefs rather than their motivational drive. In contrast, cognitive presence exerted significant positive effects on both learning motivation (*β* = 0.230, *p* < 0.001) and self-efficacy (*β* = 0.682, *p* < 0.001), highlighting its central role in enhancing both teachers’ internal drive and their confidence in instructional competence.

Further examination of the interrelationships among presences revealed that both teaching presence (*β* = 0.302, *p* < 0.001) and social presence (*β* = 0.547, *p* < 0.001) significantly predicted cognitive presence, underscoring the interdependent nature of the CoI dimensions in shaping reflective and meaningful professional learning. Overall, these findings suggest that while teaching and social presences may not directly influence professional development outcomes, they contribute indirectly through their impact on cognitive presence, learning motivation, and self-efficacy, with cognitive presence and self-efficacy emerging as the most influential predictors of teachers’ professional growth.

### 4.2 Test of mediation effects

This study employed bias-corrected nonparametric percentile bootstrap with 5,000 resamples to examine the mediating effects. The calculated indirect effects, standard errors, and 95% confidence intervals are presented in Table 6.

**Table 6:** Mediation effect test results.

Parameter	Estimate	Lower	Upper	*P*
LM ← TP	0.194	0.087	0.309	0.000
PDO ← LM	0.182	0.096	0.266	0.000
PDO ← TP	0.041	−0.046	0.121	0.345
SE ← TP	−0.022	−0.124	0.089	0.697
PDO ← SE	0.602	0.511	0.694	0.000
LM ← SP	0.065	−0.058	0.180	0.301
PDO ← SP	−0.041	−0.151	0.065	0.430
SE ← SP	0.234	0.101	0.358	0.001
LM ← CP	0.215	0.102	0.336	0.001
PDO ← CP	0.170	0.083	0.263	0.000
SE ← CP	0.599	0.488	0.716	0.000
LM ← SE	0.574	0.489	0.657	0.000
CP ← TP	0.427	0.318	0.537	0.000
CP ← SP	0.737	0.646	0.809	0.000

H13 posited that learning motivation mediates the relationship between teaching presence and professional development outcomes, while H18 proposed that self-efficacy serves as a mediator in the same relationship. The results indicate that the mediating effect from teaching presence to professional development outcomes was 0.041, *p* = 0.345, with a 95% bootstrap confidence interval of [−0.046, 0.121], which includes zero. This suggests that the mediating effect between teaching presence and professional development outcomes is not significant. Consequently, H13 and H18 are not supported, indicating that neither learning motivation nor self-efficacy mediates the relationship between teaching presence and professional development outcomes.

H14 proposed that learning motivation mediates the relationship between social presence and professional development outcomes, while H19 suggested that self-efficacy serves as a mediator in the same relationship. The results indicate that the mediating effect from social presence to professional development outcomes was −0.041, *p* = 0.430, with a 95% bootstrap confidence interval of [−0.151, 0.065], which includes zero. This indicates that the mediating effect between social presence and professional development outcomes is not significant. Consequently, H14 and H19 are not supported, suggesting that neither learning motivation nor self-efficacy mediates the relationship between social presence and professional development outcomes.

H15 proposed that learning motivation mediates the relationship between cognitive presence and professional development outcomes, while H20 suggested that self-efficacy serves as a mediator in the same relationship. The results indicate that the overall mediating effect from cognitive presence to professional development outcomes was 0.170, *p* < 0.001, with a 95% bootstrap confidence interval of [0.083, 0.263], which does not include zero, indicating a significant mediating effect. Specifically, the mediating effect of learning motivation was 0.215 (95% CI [0.102, 0.336]), and that of self-efficacy was 0.599 (95% CI [0.488, 0.716]), both excluding zero and with *p* ≤ 0.001. These findings demonstrate that both learning motivation and self-efficacy significantly mediate the relationship between cognitive presence and professional development outcomes. Therefore, H15 and H20 are supported, indicating significant mediating roles of learning motivation and self-efficacy in this relationship.

## 5 Discussion

### 5.1 Non-significant pathways of effect and the limited roles of teaching presence and social presence

The positive predictive effect of teaching presence on self-efficacy was found to be non-significant. This aligns with the notion that even a carefully designed and well-executed course does not necessarily translate into immediate or direct gains in teachers’ professional development outcomes. This finding aligns with self-efficacy theory. Bandura (1997) posited that the most influential source of self-efficacy is mastery experiences or direct personal successes. Mastery experiences—and thus the internalization of self-efficacy—occur only when learners are engaged in cognitive inquiry and practice, actively solving problems and constructing knowledge, experience, and meaning (Chen et al., 2025; Reeve, 2002). According to traditional perspectives, explicit guidance and positive feedback from teachers, as a form of verbal persuasion, are expected to enhance self-efficacy (Akcaoglu and Akcaoglu, 2022). Considering the differing findings in this study, this may be because for teachers as adult learners with a relatively high level of professional expertise, external encouragement and guidance are insufficient to influence their professional self-efficacy. They place greater value on whether they have gained tangible improvements in their capabilities through hands-on practice (Niemiec and Ryan, 2009).

The positive predictive effect of teaching presence on professional development outcomes was not significant. According to the Community of Inquiry theory (Garrison et al., 1999), the fundamental function of teaching presence lies in designing and facilitating the learning process, rather than directly producing learning outcomes. The teaching presence is the designer and facilitator of the cognitive presence and social presence. It mainly influences the outcomes of deep learning indirectly by organizing and guiding the cognitive and social processes (Yang and Lay, 2025). The key reason for this indirect relationship lies in the complexity of teacher professional development outcomes. The core of teacher professional development outcomes is the sustained change in teaching behaviors. This process of behavioral change is a complex construction involving active internalization, contextualized reflection, and integration of practice. Teaching presence constitutes the necessary external condition for this behavior to occur. However, this external condition must be transformed into personal practical knowledge through the in-depth cognitive processing of individual teachers (Gurley, 2018; Francis, 2023). Therefore, the impact of teaching presence on behavioral change is fully transmitted through the mediating chain of cognitive presence and the subsequent motivation and self-efficacy. It should be noted that although the teaching presence does not directly predict distant outcomes such as behavioral changes, it has significant predictive power for proximal results, such as significantly influencing learners’ satisfaction, their willingness to continue learning, and their perception of learning effectiveness (Joo et al., 2011; Horak, 2025). This further delineates the direct boundary of the impact of teaching presence: It is crucial for creating positive learning experiences and initiating the learning process, but transforming these experiences and intentions into solid abilities and behaviors requires more profound cognitive and psychological intermediary processes.

The positive predictive effect of social presence on learning motivation was not significant. Although social presence does not positively predict learning motivation, it can positively predict self-efficacy. The professional learning motivation of teachers is essentially practical and goal-oriented. The generation of learning motivation is more attributed to cognitive satisfaction and improvement in efficacy, rather than merely social interaction (Knowles et al., 2014). Conventional perspectives suggest that a supportive social environment can reduce anxiety, strengthen students’ confidence and self-efficacy, and thereby enhance learning motivation. Bandura’s observational learning theory (Bandura, 1997) posits that verbal persuasion and vicarious experiences are key sources of self-efficacy, which supports the notion that social presence is closely related to self-efficacy. In a supportive learning community, encouragement from peers and examples of successful practices can effectively enhance individuals’ self-efficacy. The role of social presence is not to directly “drive” things, but rather to “empower” deeper cognitive processes by creating a psychologically safe environment (Simonet et al., 2015; Luo et al., 2022). According to adult learning theory and self-determination theory, the core driver that sustains teachers’ continuous engagement stems more directly from the satisfaction of competence and autonomy needs gained through solving practical problems (Ryan and Deci, 2000; Brieger et al., 2020; Brenner, 2022; Sun and Jin, 2025). This discovery has clearly defined the precise role of social presence as a key “environmental enabler” rather than a “direct driver” in the professional development outcomes of teachers.

The positive predictive effect of social presence on professional development outcomes was not significant. A supportive community atmosphere, by itself, does not directly lead to improvements in professional competence. Some studies suggest that the primary function of social presence is to promote cognitive engagement and enhance cognitive presence, thereby indirectly influencing learning outcomes (Shea and Bidjerano, 2010). These perspectives are consistent with the pathway in the present study’s model from social presence to cognitive presence thus professional development outcomes.

### 5.2 Significant pathways of effect and the critical role of cognitive presence

Teaching presence exhibits a significant positive predictive effect on both learning motivation and cognitive presence. From this perspective, although teaching presence does not directly influence professional development outcomes or directly enhance self-efficacy, it nonetheless serves as a crucial foundation and support for igniting learning motivation and structuring cognitive inquiry activities. The effect of teaching presence on learning motivation operates through a clear course structure, meaningful task design, and timely instructional guidance, enabling learners to perceive the value of learning and their developmental trajectory, thereby activating the task-value and expectancy components highlighted in Expectancy-Value Theory (Wigfield and Eccles, 2000). The positive influence of teaching presence on cognitive presence represents one of the most central and repeatedly validated relationships within the CoI framework: without careful instructional design, facilitation, and guidance from teachers, learners are unlikely to engage in and sustain deep cognitive inquiry spontaneously (Chen et al., 2025).

Social presence exerts significant positive predictive effects on both self-efficacy and cognitive presence. It constitutes a critical condition for fostering psychological safety and enhancing confidence in professional learning. The influence of social presence on self-efficacy operates through supportive interactions within the community, offering learners peer encouragement and vicarious experiences (Bandura, 1997). Through acceptance and respect from peers, opportunities for dialog and interaction, as well as collaboration and cooperation, learners develop a stronger sense of confidence and efficacy. For teachers engaged in professional exploration and confronted with various uncertainties, social presence serves as an essential source of confidence in their professional development. A high level of social presence can effectively mitigate the personal and emotional risks associated with errors, setbacks, or feelings of embarrassment in asking questions, thereby providing a psychologically safe environment for cognition. Such an environment, in turn, creates the conditions for the emergence of more frequent and higher-order cognitive activities. Empirical evidence further indicates that social presence is particularly critical in the early stages of a course for the subsequent development of cognitive presence (Akyol and Garrison, 2008).

Cognitive presence in this model has dual attributes: it is both a direct predictor of teachers’ professional development, and also serves as an intermediary transmission mechanism that influences professional development through the aspects of teaching presence and social presence. These two roles together constitute the complete path of teachers’ professional growth within the CoI framework. At the same time, when learners successfully resolve challenging problems through cognitive inquiry, they often experience both a sense of competence and an accomplishment in performance (Chen et al., 2024). As highlighted in research on the relationship between cognitive engagement, motivation, and efficacy, successful experiences in intellectual effort and skill execution serve not only as a powerful source of self-efficacy but also as an effective catalyst for intrinsic motivation (Dadandi and Yazici, 2024; Yang et al., 2024). These findings collectively suggest that cognitive engagement exerts a positive influence on both learning motivation and self-efficacy.

### 5.3 The mediating effects of self-efficacy or learning motivation are primarily determined by cognitive presence rather than by teaching presence or social presence

Neither self-efficacy nor learning motivation exhibited significant mediating effects in the relationships between teaching presence and professional development outcomes or between social presence and professional development outcomes. The core function of social presence lies in fostering a safe, trustworthy, and open communicative environment, primarily influencing learners’ satisfaction, sense of belonging, and emotional support, rather than directly stimulating performance-oriented self-efficacy and learning motivation (Wu et al., 2023; Vansteenkiste et al., 2004; You and Kang, 2014). Prior research has indicated that community interaction among learners directly influences course satisfaction but does not have a direct effect on learning achievement (Picciano, 2002). Social presence has been found to significantly and positively predict learner satisfaction, yet its predictive power for perceived learning ability is markedly weaker than that of teaching presence (Richardson and Swan, 2003). This implies that learners’ perception of a supportive community atmosphere does not necessarily translate directly into enhanced self-efficacy or learning motivation. Teaching presence plays a structural role in learning activities, with its value residing largely in the design, organization, and guidance of the course. Such structural support is often taken for granted by learners as an environmental condition, rather than experienced as a practice that directly stimulates intrinsic motivation or self-efficacy. A plausible explanation is that, for teachers as adult learners participating in online or blended professional development programs, learning tends to be more task-oriented and cognitively driven.

Self-efficacy and learning motivation were found to exert significant mediating effects between cognitive presence and professional development outcomes. This finding is highly consistent with results reported in the mainstream literature. According to the Community of Inquiry (CoI) framework, learning emerges from the inquiry process defined by cognitive presence, which unfolds as a cyclical progression from triggering events through exploration and integration to resolution. As the core of deep learning, cognitive presence entails a series of intellectually demanding activities. When learners successfully engage in this process of cognitive inquiry, their self-efficacy is significantly enhanced; in turn, learners with high self-efficacy are more inclined to embrace cognitive challenges. Self-efficacy not only influences the selection of behaviors but also affects the level of effort and perseverance when facing challenges (Kang et al., 2024). This discovery offers significant insights for the design of teacher professional development programs: Simply providing community support or organizing teaching activities is not sufficient to trigger deep changes in motivation and efficacy; only by placing cognitive challenges at the core of the program design and allowing teachers to gain successful experiences in real exploration can the key mediator of self-efficacy be activated.

## 6 Conclusion

### 6.1 Cognitive presence is the engine for teachers’ professional growth

This study shows that the path from cognitive presence through learning motivation and self-efficacy, ultimately to professional development outcomes, constitutes the main learning approach for teachers – this discovery places cognitive presence at the core of effective professional development. Unlike teaching presence and social presence, which only play a facilitating role; cognitive presence directly promotes the enhancement of teachers’ motivation, self-efficacy and development outcomes. Deep engagement at the cognitive level represents an irreplaceable core factor in facilitating teacher professional growth. The ultimate value of all external supports, including teaching presence and social presence, lies in whether they can be successfully translated into learners’ intrinsic cognitive inquiry. This provides a practical implication for the design of teacher development programs, which should focus on how to effectively foster teachers’ cognitive presence.

### 6.2 Teaching presence and social presence are foundational, not sufficient

While these two presences do not directly determine professional development outcomes, they play indispensable roles as catalysts for cognitive activation. It should also be noted that relying solely on teaching presence and social presence, without the core element of cognitive presence, is insufficient to drive performance-oriented learning motivation and self-efficacy in teachers. Teaching presence and social presence each possess distinct psychological mechanisms and functions: teaching presence primarily acts as a guide for cognitive construction and learning motivation, whereas social presence contributes to teachers’ professional development by fostering a supportive community environment, ensuring emotional security, and enhancing self-efficacy.

### 6.3 Teachers as adult learners prioritize cognitive fulfillment over social comfort

Teacher professional development is a prototypical form of adult learning. The non-significant paths revealed in the research findings may precisely indicate that, as adult learners, teachers are primarily driven by professional development goals, with a strong emphasis on practice and a desire to enhance their intrinsic self-efficacy and learning motivation through solving authentic, context-specific problems, rather than merely benefiting from well-organized instruction or a harmonious community atmosphere. For adult learners—particularly teachers seeking professional growth—the mechanisms that stimulate learning motivation are more closely aligned with cognitive fulfillment and competence enhancement than with social interaction or emotional comfort.

## 7 Limitations and future directions

Certain limitations exist with respect to the research sample and data. The cross-sectional design precludes causal inference, suggesting the value of future longitudinal studies to test causal relationships. In addition, the reliance on teachers’ self-reports raises the possibility of social desirability effects; although efforts were made to minimize common method variance, some constraints inherent in the sample data remain unavoidable.

The evaluation of teachers’ professional development outcomes could be further diversified by incorporating socio-emotional performance indicators such as learning satisfaction and sense of community belonging. This would allow for a more in-depth examination of the multidimensional effects of social presence, for example, by investigating whether social presence exerts an indirect and sustained influence on professional development outcomes through its impact on satisfaction and community belonging.

Future research may benefit from more contextually grounded analyses, for instance by focusing on teacher groups within subject domains or developmental stages. Comparing the interaction pathways of multiple factors across such groups could yield more fine-grained insights.

This study primarily employs structural equation modeling to examine the relationships among presence, individual psychological factors, and the outcomes of teachers’ professional development, with limited attention given to qualitative data. Subsequent research could profitably incorporate qualitative approaches—such as interviews and observations—to gain deeper insight into how teachers experience the three forms of presence, and how self-efficacy and learning motivation are elicited and exert influence in the context of specific learning tasks. Such integration would provide more textured, narrative evidence to complement the quantitative findings.

## Data Availability

The raw data supporting the conclusions of this article will be made available by the authors, without undue reservation.
