# Correction: The pathways to teacher growth: mediating roles of motivation and self-efficacy within the community of inquiry framework

**DOI:** 10.3389/fpsyg.2026.1844127

**Published:** 2026-04-09

**Authors:** Mingzhang Zuo, Dengfeng Yang, Lei Xiang, Zhimiao Yang

**Affiliations:** 1 Faculty of Artificial Intelligence in Education, Central China Normal University, Wuhan, China; 2 School of Education, China West Normal University, Nanchong, China

**Keywords:** community of inquiry, learning motivation, rural teacher, self-efficacy, sense of immediacy, teacher professional development

Affiliation Faculty of Artificial Intelligence in Education, Central China Normal University, Wuhan, China was erroneously given as Faculty of Artificial Intelligence in Education, China West Normal University, Wuhan, China.

The author contributions statement was erroneously given as: MZ: Writing– original draft, Writing– review & editing. DY: Writing– original draft, Writing– review & editing. LX: Writing– review & editing. ZY: Writing– review & editing.

The correct author contributions statement is: MZ: Writing– original draft, Writing– review & editing. DY: Writing– original draft, Writing– review & editing. LX: Writing– original draft, Writing– review & editing. ZY: Writing– original draft, Writing– review & editing.

The original version of this article has been updated.

