# Supplementary material for: The pathways to teacher growth: mediating roles of motivation and self-efficacy within the community of inquiry framework
Source: Front Psychol. 2026 Mar 24;17:1755265. doi: 10.3389/fpsyg.2026.1755265 (PMC13054890; doi:10.3389/fpsyg.2026.1755265)
Supplement: Supplementary file 1 [file Supplementary_file_1.docx]

**Appendix 1 Questionnaire**

Greetings! We cordially invite you to complete an online survey based on your teacher professional development experiences. The purpose of the survey is to understand the relationships among presence, self-efficacy, learning motivation in professional learning. Your participation is completely voluntary. You are free to withdraw from the survey at any time without penalty.

Basic Information

1.Years of Teaching Experience

○ 1 year or less○ 2-5 years○ 6-10 years○ 11-20 years○ 21 years or more

2.Type of Institution

○ Primary Education○ Secondary Education○ Higher Education

3.Perceived Institutional Ranking

4.Gender

○ Male○ Female

5.Satisfaction with External Support provided for professional development

6.Primary Mode of Professional Development

○ Self-directed learning and reflection○ Peer collaboration or professional learning communities○ Teacher development schools or training programs○ Routine teaching research and practice○ Guidance from experts or master teachers

7.Most Effective Format Based on Your Experience

○ Face-to-face training/workshops/teaching research○ Online learning/training/workshops○ Blended (online and face-to-face) format

8.Satisfaction with Personal Professional Development Outcomes

Teaching Presence

1.The instructor clearly defined or communicated important goals for teacher professional development.

2.The instructor provided effective guidance on how to participate in discussions and inquiry activities.

3.The instructor helped me distinguish between feasible and unfeasible aspects of the development goals, thereby facilitating my progress.

4.The instructor encouraged me to explore new phenomena or issues in the field of teaching.

5.The instructor helped maintain focus on key topics and sustain productive dialogue.

6.The instructor strengthened the sense of communication and collaboration among peers.

7.The instructor clearly communicated important dates or deadlines for learning activities.

8.The instructor provided constructive feedback on my learning process and outcomes.

Social Presence

1.I felt like a welcome member of the group during interactions with my peers.

2.I felt comfortable trusting other peers even when disagreeing with them.

3.I felt that my viewpoints were trusted and respected by other peers.

4.Interactions with other members enhanced my sense of belonging in the teaching profession.

5.I found the online (or web-based) communication platform to be an excellent medium for interaction.

6.Discussions with peers helped me develop a stronger sense of collaboration.

7.I was able to form distinct impressions of some fellow participants.

Cognitive Presence

1.Frequently posed questions related to the training theme to stimulate deeper thinking.

2.Was able to apply the acquired knowledge to teaching or other non-teaching contexts.

3.Constructed explanations or solutions for teaching-related problems through the learning process.

4.Grasped fundamental concepts and diverse perspectives through thematic study, reflection, and discussion.

5.Engaged in critical examination of my own and others' viewpoints, seeking evidence for support.

6.Could clearly articulate the learned knowledge and describe how to test it in practice.

7.Proactively integrated information and perspectives from various sources to develop a personal understanding of issues.

Learning Motivation

1.I found the learning tasks interesting.

2.I considered the learning activities valuable.

3.I did not feel nervous at all when participating in the learning activities.

4.I believed the related activities facilitated understanding of the learning content.

5.I found the material to be learned relatively easy.

6.I liked the learning content very much.

7.I was satisfied with my learning performance.

8.I felt that participation in the learning was very helpful to me.

Self-Efficacy

1.I am confident in handling unexpected situations during learning.

2.If I invest sufficient effort, I can resolve most problems encountered in learning.

3.I can remain calm when facing difficulties in learning, as I believe in my ability to solve them.

4.When encountering learning problems, I can usually identify multiple solutions.

5.Even when facing challenging learning problems, I can typically devise a solution.

6.No matter what occurs during learning, I can handle it with ease.

Teacher Professional Development Outcomes

1.I feel confident in managing complex classroom situations.

2.I am confident in my ability to design innovative teaching activities.

3.I hold a firm belief in promoting student development.

4.Overall, my teaching is conducted with ease and is highly effective.

5.I frequently engage in self-assessment after teaching to review successes and shortcomings.

6.I tend to analyze teaching issues or effectiveness based on deep educational theories rather than superficial phenomena.

7.I proactively seek feedback from students, colleagues, or mentors to reflect on my teaching.

8.I consciously adjust and refine my teaching based on reflective outcomes.

9.In instructional design, I prefer independent thinking over complete reliance on teaching references.

10.I often take the initiative to experiment with new teaching methods or technologies.

11.I am capable of setting clear, long-term professional development goals for myself.

12.In the past year, I have experimented with innovative instructional models such as project-based learning or the flipped classroom.

13.I have attempted to use various digital tools, including big data, learning analytics, and AI-powered tools.

14.I adapt or innovate teaching materials to meet student developmental needs.

15.I actively apply knowledge gained from training sessions into classroom teaching.
